# Non-medical costs incurred by critically ill patients with dengue, sepsis and tetanus within a major referral hospital in Southern Vietnam: a cost of illness study

**DOI:** 10.1136/bmjph-2024-002169

**Published:** 2025-08-20

**Authors:** Trinh Manh Hung, Thanh Nguyen Nguyen, Mau Toan Le, Phuc Hau Nguyen, Thanh Phong Nguyen, Thi Hue Tai Luong, Buu Chau Le, Ba Thanh Pham, Thi Trang Khiet Tieu, Thi Diem Thuy Tran, Minh Yen Lam, Sophie Yacoub, Sayem Ahmed, Louise Thwaites, on behalf of the VITAL Consortium, Dang Phuong Thao, Hugo C Turner

**Affiliations:** 1 Oxford University Clinical Research Unit, Ho Chi Minh City, Viet Nam; 2 Hospital for Tropical Diseases, Ho Chi Minh City, Viet Nam; 3 Nuffield Department of Medicine, University of Oxford Centre for Tropical Medicine and Global Health, Oxford, UK; 4 Health Economics and Health Technology Assessment, School of Health and Wellbeing, University of Glasgow, Glasgow, UK; 5 MRC Centre for Global Infectious Disease Analysis, School of Public Health, Imperial College London, Norfolk Place, London, UK

**Keywords:** economics, Disease Vectors, Health Services Accessibility

## Abstract

**Introduction:**

Improving the knowledge of the costs of critical care is vital for informing health policy. However, cost data remain limited, particularly for low- and middle-income countries. The aim of this cross-sectional study is to describe the direct/indirect non-medical costs incurred by critically ill tetanus, sepsis and dengue patients and their families during their hospitalisation, using data from a major referral hospital in Vietnam.

**Methods:**

This study was conducted within the Hospital for Tropical Diseases in Ho Chi Minh City, a tertiary referral hospital specialising in infectious diseases serving Southern Vietnam. Patients who were admitted to the intensive care unit (ICU) and diagnosed with either tetanus, dengue or sepsis were enrolled between April and November 2022. In total, 94 patients (and their caregivers) were interviewed. Structured questionnaires were used to estimate the direct non-medical costs and indirect costs (costs related to productivity/time losses) incurred during their hospitalisation by the patients and their caregivers (ie, the patients’ perspective).

**Results:**

Overall, the estimated median total direct/indirect non-medical costs of the sample varied between US$511 and US$814 per patient, depending on the approach used to value the indirect costs. These total costs were broadly similar among sepsis and tetanus cases, but lower for dengue cases. The estimated indirect costs were highly sensitive to the approach used to monetise productivity losses and the valuation of informal care.

**Conclusion:**

This study demonstrates that patients admitted to the ICU with a severe infection of these diseases can incur notable direct/indirect non-medical costs. These results highlight the importance of further research in this area. These findings are particularly relevant in the context of universal health coverage targets, as even with 100% coverage of medical costs, many families are still likely to suffer financial hardship.

**WHAT IS ALREADY KNOWN ON THIS TOPIC**

- Critical care services are one of the most expensive healthcare services globally. While some studies have described the direct medical costs of critically ill patients, data related to the direct non-medical and indirect costs they and their families incur are more limited, particularly for low- and middle-income countries.

**WHAT THIS STUDY ADDS**

- This study demonstrates that critically ill tetanus, sepsis and dengue patients and their caregivers can incur notable direct and indirect non-medical costs.

**HOW THIS STUDY MIGHT AFFECT RESEARCH, PRACTICE OR POLICY**

- This study highlights the importance of evaluating the direct non-medical and indirect costs associated with critically ill patients. These findings are relevant for research and policy, particularly in the context of universal health coverage targets, as even with very high coverage, many families of critically ill patients will likely continue to suffer significant financial hardship due to non-medical costs. This highlights the important need for further work in this area.

## Background

 Making progress towards universal health coverage (UHC), ensuring that people have access to the healthcare they need without suffering financial hardship, is a policy priority for many countries, as well as key stakeholders, such as the WHO and World Bank.^1^,^4^ Improving the knowledge of the costs of different types of healthcare is vital in this context for informing resource utilisation and health policy. However, certain areas have less cost data.

Critical care is the process of looking after patients who either suffer from life-threatening conditions or are at risk of developing them and is needed for a range of different aetiologies.^5^ Critical care remains particularly limited in low- and middle-income countries (LMICs) and critical illness results in millions of deaths globally each year.^6^,^9^ The proportion of critical care admissions and deaths related to infectious diseases is likely to be higher in LMICs.

The health economic literature in this area is limited, with the majority of the available studies focusing on the direct medical costs incurred (ie, often related to the costs on the patients’ medical bills).^610^,^14^ These have shown that critical care is expensive relative to typical health spending.^615^,^18^ However, there will be additional non-medical costs incurred by the patients and their families beyond these medical costs^19^,^21^—including direct non-medical costs, the costs related to the consumption of non-medical resources (such as transportation), and indirect costs related to the productivity losses of patients and their informal caregivers (also known as productivity costs). These non-medical costs are particularly relevant to critical care, as critically ill patients have longer stays in the hospital and informal caregivers can play an important role in their care.^22 23^ Crucially, even patients that have high health insurance cover will incur these costs and they need to be considered to get a full picture of the costs being incurred. Having an understanding of these costs is important from an equity standpoint, as poorer groups of the population are most at risk of suffering financial hardship. Despite their importance, information related to the burden incurred by informal caregivers is a particularly neglected area^24 25^ but is very pertinent to LMICs.^26^

In Vietnam, informal caregivers play an important role in taking care of patients both in hospital and at home. Studies have found that the burden of informal caregivers is high in Vietnam and highlighted issues around the lack of community resources, economic impact and low quality of life.^27^,^29^ Interestingly, many caregivers did not consider caring as a burden but expressed their suffering as exhaustion or sadness.^29^ However, most of the published studies were related to chronic diseases or cancer, and very limited information is available related to informal caregivers of severely ill patients, particularly with infectious diseases. In addition, to the best of our knowledge, no studies have quantified the cost of caring for severely ill hospitalised patients.

This study aimed to investigate the direct/indirect non-medical costs incurred by critically ill tetanus, sepsis and dengue patients during their hospitalisation at a major referral hospital in Vietnam. The study also aimed to capture the costs incurred by the patient’s informal caregivers, who can play a crucial role in caring for hospitalised patients in Vietnam but for which limited information is available, particularly for those taking care of severely ill patients.^30^ In addition, we investigated the impact of using a range of approaches for quantifying indirect costs and valuing informal caregivers’ time—which despite being key cost drivers, their calculations are often inconsistent and poorly reported. The study focused on tetanus, sepsis and dengue as they are responsible for the majority of patient admissions to the intensive care unit (ICU) within the sampled hospital.^31^ The overarching goal of this study is to act as proof of principle regarding the potential importance of these costs for severe infections.

## Methods

This costing study was conducted within the Hospital for Tropical Diseases (HTD) in Ho Chi Minh City (HCMC). This setting was chosen because it is the only referral hospital dedicated to the treatment of infectious diseases in HCMC and serves patients from a wide geographical area across the south of Vietnam including Mekong Delta Region, central highland and Southeast Region. Specifically, HTD is the highest-level hospital to treat severely ill patients with infectious diseases in Southern Vietnam. This makes it a good setting for sampling critically ill patients.

### Patient and caregiver enrolment and data collection

The sample size of this study was estimated using the guidance from Johnston *et al* study;^32^ using a probability of type 1 error of 15%, and coefficient of variation of 0.73 (informed by a previous study^10^), the estimated sample size was 91. Patients were screened and recruited if they were admitted to the adult ICU, diagnosed with either dengue, sepsis or tetanus, and stayed at the ICU for at least 24 hours to be eligible for recruitment (no other eligibility criteria were applied). Patients and their informal caregivers were recruited at the ICU and tracked until they were discharged from the hospital. Patients who were transferred to the general wards after the ICU were also tracked (these data were also collected—table 1).

**Table 1:** Outline of the types of data collected

	Patients	Informal caregivers
Baseline characteristics	Patients were asked questions related to their key characteristics (including age, gender, address, employment status etc.).	Informal caregivers were asked questions related to their key characteristics (including age, gender, employment status etc.).
Direct non-medical costs: food	The amount of money spent on the patient’s food per day (additional to prescribed nutrition) in the ICU and the general ward was collected (when possible, this information was retrieved from their informal caregivers). This was multiplied by the number of days the patient was hospitalised to get a total. Any free food received from charities was not included.	The amount of money spent on the informal caregivers’ food per day in the ICU and the general ward was collected. This was multiplied by the number of days the caregivers spent at the hospital to get a total. Any free food received from charities was not included.
Direct non-medical costs: travel	The costs related to the patient’s travel to the hospital and their travel back home were collected.*	The costs related to the informal caregivers’ travel on the first day they visited the patient and their travel back home were collected.†‡
Direct non-medical costs: other	Patients were asked if they incurred any costs related to childcare arrangements.	Informal caregivers were asked if they incurred any costs related to accommodation.§
Indirect costs (productivity costs)	The patients’ productivity losses were quantified based on the number of days they stayed at the hospital. These were monetised based on the approaches outlined in online supplemental Table S1, Table S2 and Box S1.	The informal caregivers’ productivity losses were quantified based on the number of hours they reported spending at the hospital per day multiplied by the number of days they spent with the patient (this was divided by 24 to convert the productivity losses into a daily value). These were monetised based on the approaches outlined in online supplemental Table S1, Table S2 and Box S1.
Coping strategies	Patients were asked questions regarding how they would pay for the hospitalisation and how much money they would have to borrow.	Not applicable.

* If the patient did not know the cost related to their travel back home from the hospital (and if they consented), they were contacted via phone after they were discharged.

† If a caregiver was not available at the time of the interview, another caregiver of the patient was asked on their behalf.

‡ It was not possible to capture the additional travel-related costs if informal caregivers travelled back and forth multiple times.

§ Hospital policy allows for one space of overnight accommodation for a family member when a patient is in the ICU. In addition, while a patient is in the general ward, one caregiver can stay overnight with them in the ward.

ICU, intensive care unit.

Owing to the complexity of tracking/gathering information from patients hospitalised in the ICU, we used a convenience sampling approach to enrol the participants. Enrolment was conducted between April and November 2022. Informal caregivers were defined as those who visited and looked after the patient for at least 2 hours a day. All informal caregivers of the enrolled patients were recruited when they first stayed in the hospital and were carefully tracked during the time in the ICU and/or the general wards. If an informal caregiver was unavailable for an interview, their information was obtained from another caregiver.

The patients and their caregivers were given structured questionnaires to collect the required data including their demographic characteristics, the costs incurred and their coping strategies (table 1). Patients and caregivers were interviewed within 24 hours before their hospital discharge to capture cost information for their total stay at the ICU and the general ward to minimise potential stress to the interviewee. It should be noted that the direct non-medical and indirect costs were estimated for this hospitalisation period only.

### Patient and public involvement

Patients and their informal caregivers were not involved in the study design, conduct, report and dissemination of this study.

### Cost analysis

The direct/indirect costs incurred by the patients and their caregivers were estimated from the patient’s perspective using the data obtained from the structured questionnaires (table 1). Direct medical costs (costs related to the use of medical resources/goods) were not included, as they were considered outside the scope of this study. All costs were presented in 2021 US$ prices using the exchange rate of US$1 to 23 160 dong.^33^

Using the data from the overall sample (ie, not stratifying by disease), a multiple linear regression analysis was used to explore the association between the total direct/indirect non-medical cost and other important variables. Owing to limited observations, we included the following variables: number of days in the ICU, number of days in the general wards, the address of the patients and number of informal caregivers across the five indirect cost calculation scenarios (defined in online supplemental Table S1) as described with the following formula:


$$\begin{array}{l} \;Ln\left(Total\;non\;medical\;cost_{i}\right)=\alpha +\beta_{1i}*Gender+\beta_{2i}*Address\\ \;+\beta_{3i}*ICU\;days+\beta_{4i}*General\;Ward\;days\\ \;+\beta_{5i}*Number\;of\;cargivers \end{array}$$

where ln() is the natural logarithm, total cost_*i*_ is the total cost estimated within scenario *i*, *α* is the intercept, and *β*_1*i*_, *β*_2i_, *β*_3*i*_, *β*_4*i*_ and *β*_5*i*_ are parameters estimated within scenario *i*.

We only valued the productivity losses of patients and their informal caregivers incurred during the patient’s hospitalisation. Productivity losses after discharge or those related to mortality were not included in this analysis. Due to the variation surrounding indirect cost calculations and lack of consensus regarding the correct methodology,^34^,^37^ we used five different methodology scenarios for the indirect cost calculation—summarised in online supplemental Table S2 (further information in online supplemental Table S1). These scenarios used different assumptions/wage sources, allowing us to compare the impact of using different methods for monetising productivity losses on the cost estimates. Note that these calculations were based on lost days or lost hours due to the ICU admission and not only based on work time lost. The approaches were in line with the human capital approach, in which all potential production not performed by a person was counted as a production loss.^38^

The costs were stratified based on whether they were incurred by the patient versus their informal caregiver(s) and whether they were incurred in the ICU versus the general ward. The total costs presented accounted for the proportion of patients that were directly discharged from the ICU and did not go to the general ward.

## Results

### Baseline information of patients and informal caregivers

#### Patients

The families of 110 eligible patients were approached for enrolment, of whom 100 agreed to participate. Six patients were lost to follow-up owing to being transferred to other hospitals or asking to go home. Thus, data were collected from 94 patients and their corresponding informal caregivers (204 people). Data related to the baseline information of the patients are presented in table 2. The patients had a median age of 42 years and mostly lived outside HCMC (63.83%). The majority of patients (72.34%) reported being covered by the government’s health insurance programme, and no patients reported using private health insurance. The patients spent a median of 10.5 days (IQR: 5.00–22.80) in the ICU and 5.50 days (IQR: 4.00–8.25) in the general ward. These characteristics were influenced by their specific disease, with tetanus patients being older, more likely to live outside of HCMC and having a longer stay in the ICU.

**Table 2:** Basic characteristics of sampled ICU patients

	Dengue (n=44)	Sepsis (n=12)	Tetanus (n=38)	Overall (N=94)
Gender				
Female, n (%)	27 (61.36%)	5 (41.67%)	10 (26.32%)	42 (44.68%)
Male, n (%)	17 (38.64%)	7 (58.33%)	28 (73.68%)	52 (55.32%)
Address				
Ho Chi Minh City, n (%)	22 (50.0%)	5 (41.67%)	7 (18.42%)	34 (36.17%)
Other provinces, n (%)	22 (50.0%)	7 (58.33%)	31 (81.58%)	60 (63.83%)
Age (years)				
Median (Q1, Q3)	30.00 (21.75, 39.75)	50.50 (43.25, 60.00)	58.00 (42.50, 66.00)	42.00 (29.25, 59.00)
Governmental health insurance status				
No, n (%)	17 (38.64%)	0 (0.00%)	9 (23.68%)	26 (27.66%)
Yes, n (%)	27 (61.36%)	12 (100.00%)	29 (76.32%)	68 (72.34%)
Length of stay in the ICU (days)				
Median (Q1, Q3)	5.00 (4.00, 7.50)	10.50 (5.50, 22.50)	21.50 (13.00, 30.00)	10.50 (5.00, 22.75)
Where the patients went after ICU				
Number discharged directly from the ICU, n (%)	5 (11.36%)	2 (16.67%)	8 (21.05%)	15 (15.96%)
Number transferred to the general ward, n (%)	39 (88.64%)	10 (83.33%)	30 (78.95%)	79 (84.04%)
Number of days in the general wards				
Median (Q1, Q3)	4.00 (2.00, 6.00)	9.50 (7.00, 10.75)	7.00 (5.00, 9.00)	6.00 (4.00, 8.50)
Number of days in the hospital				
Median (Q1, Q3)	9.50 (6.75, 15.50)	20.00 (13.75, 33.75)	25.50 (18.25, 37.25)	17.0 (10.00, 28.75)
Employment status				
Full time job, n (%)	10 (22.73%)	2 (16.67%)	12 (31.58%)	24 (25.53%)
Looking after home, n (%)	4 (9.09%)	2 (16.67%)	1 (2.63%)	7 (7.45%)
Own business/farming, n (%)	14 (31.82%)	1 (8.33%)	5 (13.16%)	20 (21.28%)
Part time job, n (%)	2 (4.55%)	0 (0%)	6 (15.79%)	8 (8.51%)
Retired, n (%)	2 (4.55%)	4 (33.33%)	10 (26.32%)	16 (17.02%)
Student, n (%)	7 (15.91%)	1 (8.33%)	0 (0%)	8 (8.51%)
Unemployed, n (%)	3 (6.82%)	0 (0%)	0 (0%)	3 (3.19%)
Other, n (%)	2 (4.55%)	2 (16.67%)	4 (10.53%)	8 (8.51%)
Method of transportation to the hospital				
Ambulance, n (%)	33 (75.00%)	10 (83.33%)	26 (68.42%)	69 (73.40%)
Hired motorbike/own motorbike, n (%)	6 (13.63%)	1 (8.34%)	2 (5.26%)	9 (9.57%)
Taxi/hired car, n (%)	4 (9.09%)	1 (8.33%)	9 (23.68%)	14 (14.89%)
Other, n (%)	1 (2.28%)	0 (0%)	1 (2.64%)	2 (2.13%)

Q1: 25% of IQR and Q3: 75% of IQR.

ICU, intensive care unit.

#### Informal caregivers

Each patient had a median of two different informal caregivers over the course of their hospitalisation. A summary of the baseline characteristics of these informal caregivers is presented in online supplemental Table S3. Their median age was 41 years, and a higher proportion were female (56.86%). The majority were close relatives (a child, parent or spouse) of the patient. In terms of occupation, around 33.82% of the caregivers reported being in full-time employment and 29.41% reported being self-employed/in informal employment. 58.82% travelled to the hospital with the patient and the most common method (46.08%) was an ambulance.

### Direct non-medical costs

The direct non-medical costs are summarised in table 3. Generally, most patients (73.40%) used an ambulance on the day they were admitted to the hospital, either from their home or while being transferred from another hospital. The median travel-related cost incurred by the patients was US$60.45 (IQR: 6.48–140.32). However, these costs were notably different between patients from HCMC (US$4.71) and patients from outside HCMC (US$98.55) (online supplemental figure S1). This was the main driver in the variation in travel-related costs across the different diseases (table 3 and online supplemental figure S1). The additional travel-related costs incurred by the patients’ informal caregivers were smaller, with a median of US$12.52 (IQR: 0–44.80). The total median travel-related cost incurred by the patients and caregivers was US$79.02 (IQR: 13.44–187.82) per patient.

**Table 3:** Direct non-medical costs incurred by the patients and informal caregivers (2021 US$ prices)

Median (Q1; Q3)	Dengue (n=44)	Sepsis (n=12)	Tetanus (n=38)	Overall (N=94)
Transportation costs				
Patient	24.29 (3.13; 82.09)	54.94 (4.58; 112.42)	82.04 (28.07; 188.36)	60.45 (6.48; 140.32)
Caregivers	7.23 (0.00; 27.42)	11.01 (1.94; 29.47)	32.38 (6.10; 61.37)	12.52 (0.00; 44.80)
Food costs in ICU				
Patient	10.36 (0.00; 21.59)	0.00 (0.00; 17.10)	0.00 (0.00; 0.00)	0.00 (0.00; 14.90)
Caregivers	25.91 (10.58; 59.37)	38.86 (16.19; 60.45)	68.01 (48.36; 114.85)	45.34 (20.29; 92.83)
Food costs in general ward				
Patient	21.59 (11.01; 33.03)	38.86 (18.13; 64.77)	34.54 (22.67; 48.58)	26.99 (17.27; 43.18)
Caregivers	17.27 (5.40; 32.38)	34.54 (16.19; 50.73)	25.91 (14.68; 38.64)	22.67 (10.79; 38.64)
Total food cost				
Patient	32.38 (13.98; 47.42)	37.82 (9.72; 77.18)	32.49 (15.98; 42.96)	32.82 (14.09; 48.90)
Caregivers	58.29 (25.91; 84.52)	85.49 (33.46; 109.02)	95.10 (64.98; 139.90)	67.36 (33.41; 124.03)
Accommodation cost				
Patient	NA	NA	NA	NA
Caregivers	2.16 (1.73; 4.21)	4.53 (2.37; 9.72)	9.28 (5.61; 12.95)	4.97 (2.16; 10.25)
Total direct non-medical cost				
Patient	56.78 (21.59; 119.01)	79.88 (9.72; 171.20)	98.45 (41.23; 179.19)	74.59 (32.35; 154.77)
Caregivers	70.49 (33.14; 109.67)	100.82 (41.77; 146.10)	127.16 (75.78; 197.32)	90.89 (52.14; 153.55)

Q1: 25% of IQR and Q3: 75% of IQR.

Further details on the data collected are provided in table 1.

The total cost accounts for the proportion that was not transferred to the general ward.

NA, not applicable.

The food-related costs incurred by patients were mostly trivial while in the ICU, but higher while they were in the general ward, with an overall median of US$26.99 (IQR: 17.27–43.18). On average, the patients’ total related food cost was US$32.82 (IQR: 14.09, 48.90). In contrast, the total median food-related costs incurred by their caregivers were US$67.36 (IQR: 33.41–124.03) over the whole course of the patient’s hospitalisation, US$45.34 of this was incurred while the patient was in ICU. The total median food-related cost incurred by the patients and caregivers was US$97.58 (IQR: 58.68–167.04) per patient.

The other types of direct non-medical costs were not found to be notable. For example, accommodation-related costs were relatively low with a median of only US$4.97 (IQR: 2.16–10.25) per patient and no patients reported incurring any direct costs for childcare arrangements.

Overall, the total median direct non-medical cost including the caregiver’s costs was US$169.58 (IQR: 104.44–362.37) per patient (online supplemental Table S4). US$74.59 of this was incurred by the patients and US$90.89 by their informal caregivers (table 3). The total direct non-medical cost varied among the different diseases and was lowest for dengue with a median of US$130.48 (IQR: 83.44–277.96) and highest for tetanus with a median of US$235.86 (IQR: 146.55–398.21) (online supplemental Table S4).

### Productivity losses and indirect costs

**Figure 1:**
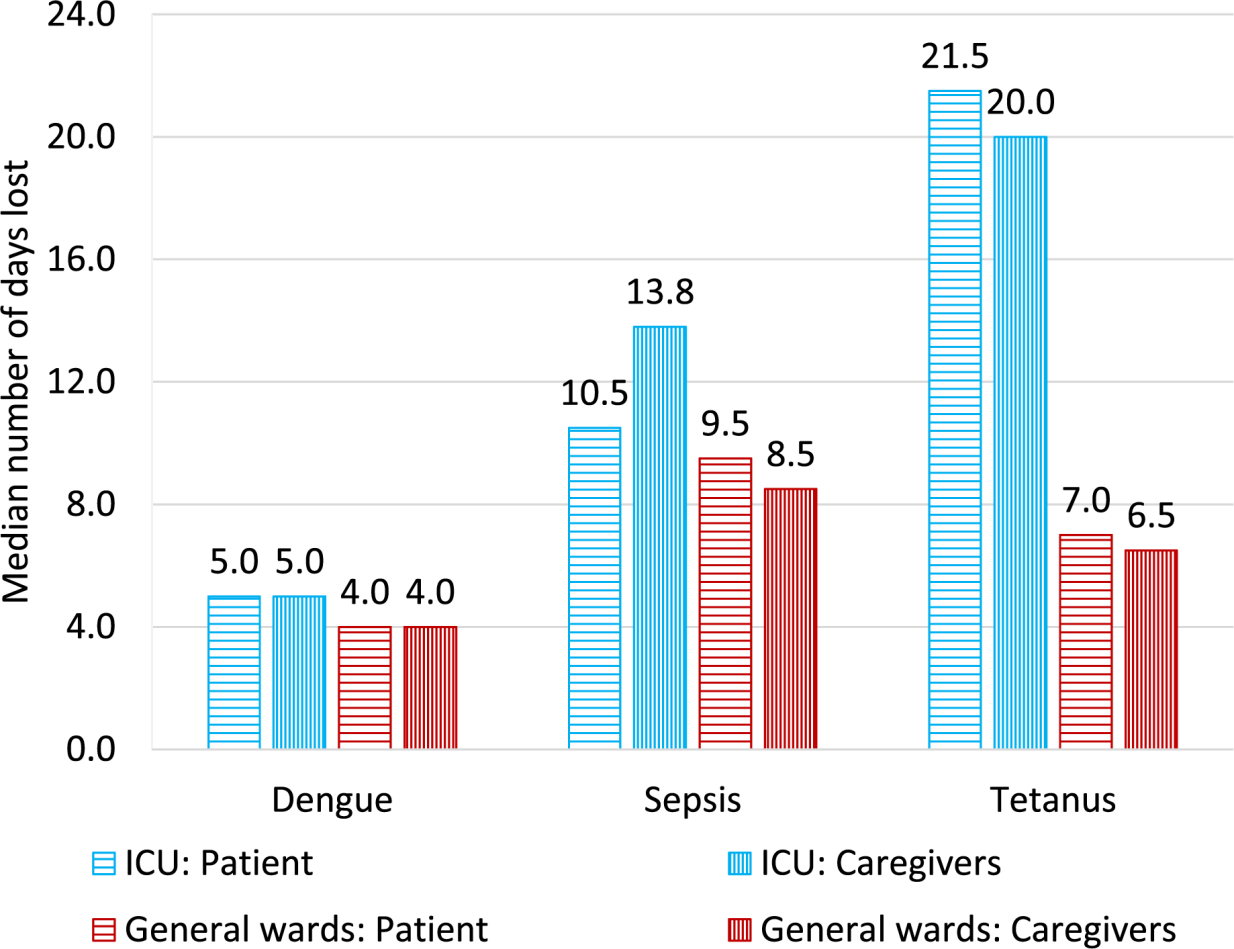
The productivity loss of the patients and their informal caregivers**. **IQRs for these data are presented in online supplemental Tables S2 and S3. ICU, intensive care unit.

The patients’ and caregivers’ productivity losses presented as the number of days lost are outlined in figure 1. The majority of patients spent longer in the ICU than in the general ward. Overall, the median number of days the patients spent hospitalised was 17.00 (IQR: 10.00–28.75). In contrast, the patients’ informal caregivers spent a median total of 19.00 days (IQR: 9.72–34.00) caring for them over the hospitalisation period. However, both of these varied according to the disease in question, with the number of lost days being higher for caregivers of patients with tetanus and lower for those caring for patients with dengue (figure 1).

The estimated indirect costs were highly sensitive to the approach used to monetise the productivity losses (online supplemental Tables S5 and S6). The lowest values corresponded to scenario 1, using the minimum wage for everyone regardless of employment status. In contrast, the highest values occurred when using scenario 4 where the productivity losses were valued based on the per capita gross domestic product (GDP) and scenario 3 where each hour the informal caregivers spent with the patient was valued. It is notable that for the informal caregivers, their estimated productivity cost varied significantly depending on if their time was valued on a daily basis (ie, each lost day being valued based on 8-hour wage) or if their time was valued on an hourly basis like scenario 3 presented in online supplemental Table S6.

Overall, the median total direct and indirect non-medical costs including the caregiver’s costs varied from US$511.33 to US$813.81 per patient during their hospitalisation depending on the approach used to value the indirect costs (table 4). When using GDP per capita to value the indirect costs (scenario 4), the total median direct and indirect non-medical cost was US$473.68 (IQR: 304.84–937.17) for dengue, US$1121.49 (IQR: 973.92–1466.86) for sepsis and US$1046.12 (IQR: 760.27–1383.47) for tetanus.

**Table 4:** Total direct/indirect non-medical cost incurred by the patients and informal caregivers (2021 US$ prices)

	Dengue (n=44)	Sepsis (n=12)	Tetanus (n=38)	Overall (N=94)
	Median (Q1; Q3)	Median (Q1; Q3)	Median (Q1; Q3)	Median (Q1; Q3)
Scenario 1: all productivity losses valued based on the minimum wage.	295.82 (202.66; 617.71)	668.45 (517.39; 829.02)	621.98 (468.43; 912.51)	511.33 (282.84; 767.49)
Scenario 2: productivity losses for patients and caregivers in full-time employment valued based on their reported wages. For those not in full-time employment, productivity losses were valued based on the daily minimum wage.	326.39 (226.02; 619.75)	837.54 (619.18; 1075.08)	732.03 (498.70; 1063.22)	552.39 (309.78; 901.06)
Scenario 3: productivity losses for patients in full-time employment were valued based on their reported wages. For those not in full-time employment, the minimum wage was used. The productivity losses of the caregivers were valued based on an hourly minimum wage (considering each hour they spent with patient).	430.68 (308.33; 778.10)	1048.90 (718.23; 1365.43)	1037.15 (744.83; 1516.03)	754.15 (425.26; 1295.46)
Scenario 4: productivity losses valued based on the per capita GDP for both patients and informal caregivers.	473.78 (304.84; 937.17)	1121.49 (973.92; 1466.86)	1046.12 (760.27; 1383.47)	813.81 (462.32; 1283.01)
Scenario 5: productivity losses for those in formal employment valued based on the average national wage and productivity losses for those not in formal employment were valued based on the daily minimum wage.	322.92 (227.69; 656.83)	779.17 (567.13; 935.01)	720.76 (522.78; 1146.43)	567.72 (313.05; 926.44)

Further description related to the different scenarios for the valuation of the indirect costs is presented in online supplemental Table S1.

GDP, gross domestic product.

The median total cost per patient per day varied from US$27.67 to US$44.71 depending on the approach used for the indirect cost calculation (online supplemental Table S7). These per day values were more consistent across the diseases. The total cost per day when using the GDP per capita valuation approach was US$46.01 (IQR: 38.11–54.85), US$47.21 (IQR: 34.35–53.17) and US$37.74 (IQR: 34.69–47.01) for dengue, sepsis and tetanus, respectively.

A univariate analysis revealed a positive linear association between the total cost and the length of stay in the hospital (online supplemental figure S2). In addition, the multiple linear regression analysis identified that the length of stay in the ICU, length of stay in the general ward and residing outside of HCMC were statistically significant predictors of total non-medical cost across all indirect cost calculation scenarios (online supplemental Table S8).

### Financial coping strategies of the patients

Beyond the non-medical costs incurred, we also found that there are broader impacts of the ICU burden on the patient/household. Among the sampled 94 patients, 55 (58.51%) reported having to borrow money or sell assets due to their hospitalisation (online supplemental Table S9). This would also be to cover the copayment of the medical bill and not just the costs shown here. Most of these patients (48/55) borrowed from family or friends, four patients needed a private loan (ie, from a non-bank lender) and three patients needed to sell their property/possessions. The median amount of money these patients borrowed was US$1295.34 (IQR: 863.56–2158.89) (online supplemental Table S9). This amount is double the sample’s reported monthly family income with a median of US$604.49 and triple the reported monthly household expenditure with a median of US$431.78 (online supplemental Table S10). The median amount borrowed was similar for those with and without insurance. However, the range in the amount borrowed was higher for those without insurance (online supplemental figure S3).

## Discussion

Although data on the costs of critical care for severe infections remain limited, it is known that stays in ICUs are associated with notable direct medical costs.^610^,^14^ This study further demonstrates that critically ill patients and their families can also incur notable direct and indirect non-medical costs.^19^,^21^ This highlights the importance of valuing unpaid work, particularly in the context of critical care, and the need for more attention to this area.

The estimated median total direct and indirect non-medical cost varied between US$511.33 and US$813.81 per patient (including the caregiver’s costs) depending on the approach used to value the indirect cost. On average, these costs were higher for patients with tetanus or sepsis and lower for patients with dengue (table 4). This is significant in the context of Vietnam, and for comparison, the monthly minimum wage ranges between US$140 and US$202.^39^

In addition, in order to understand the full picture of the costs of critical care, it is important to also consider direct medical costs. A previous study, implemented on the same diseases and same setting, found that the overall median direct medical cost for ICU patients was US$63.4 (IQR: 24.8–98.0) per day.^10^ In comparison, the average non-medical cost from this study was US$27.67 (IQR 20.48–33.57) per day when using the most conservative indirect cost scenario. While these costs vary by the specific disease and patient severity, these results emphasise that even though direct medical costs are typically the main driver in critical care costs, non-medical costs remain a significant component and can contribute significantly to the overall cost.

Of our sample, 58.51% reported having to borrow money or sell assets due to their hospitalisation. It should be noted that this would also be for the copayment of their medical bill and not just the costs shown here. This highlights the importance of further investigating the burden of the financial consequences and coping strategies associated with critical care. A study by Prinja *et al*^19^ investigating the costs associated with liver disorders in India reported a similar pattern of coping mechanisms, with 47% of the sampled ICU patients needing to borrow money to cover their expenses.

### Generalisability and representativeness of the sample

It is important to note that this study purposely investigated critically ill patients with either tetanus, sepsis or dengue who were admitted to HTD in HCMC, Vietnam. Notably, HTD is a major referral hospital that accepts referrals from both self-presenting patients and patients from other healthcare facilities from a catchment area covering Southern Vietnam, which has a combined population of approximately 40 million people.^40^ A significant proportion of severe cases of these diseases in Southern Vietnam would be sent to HTD. A previous study by Thuy *et al*^31^ describing adult ICU cases at HTD between November 2014 and January 2016 reported that 81% of their sample (296/364) related to these diseases. Although the exact unit cost estimates we present are not necessarily directly generalisable to ICU admissions for other conditions, they do demonstrate the potential magnitude of non-medical costs in Vietnam.

Furthermore, a convenience sampling approach was used to enrol the patients due to the complexity of tracking/gathering information from severely ill patients. This could lead to biases in the results. However, we found that our sample of patients with these diseases provided a good representation in terms of the age range and rural versus urban residents for patients at HTD. In addition, the median duration of ICU admission of 10 days reported for Thuy *et al*’s^31^ sample of 364 ICU patients at HTD was highly consistent with the 10.5 days observed from this study. This indicates that our sample was representative of ICU patients at HTD. The relative distribution of the three diseases among the sample in this study varied from that observed by Thuy *et al*,^31^ with this study having a higher proportion of dengue cases. It should be noted that the contribution of each disease may vary depending on various factors and these proportions will not necessarily be consistent across different years. For example, this will be heavily influenced by the variation in yearly incidence of these diseases, particularly dengue. Due to this, our sample size for sepsis was lower compared with the other diseases.

The mean household income of this sample was US$673.61 (online supplemental Table S8). With a mean household size of 3.78, the resulting per capita income was US$178. This is consistent with the monthly average income per capita for Vietnam (US$181.5) reported by the General Statistics Office of Vietnam for 2021.^41^

### The patients’ informal caregivers

Informal caregivers were defined as those who visited and looked after a patient for at least 2 hours a day. On average, each patient had two different informal caregivers during their hospitalisation—each spending on average 19 hours a day with the patient while in the ICU and 14 hours a day while in the general ward (online supplemental Table S3). In general, data on the characteristics of informal caregivers are limited within the literature. An analysis of the economic value of non-professional care across Europe^42^ which did not focus on critical care reported similar characteristics related to caregiver age (52 vs 41 years old) and the proportion that was female (61% vs 57%). However, there was a difference in the employment status of the caregivers—with a higher proportion being retired than we observed (24% and 4%). There was also notable variation across the countries included, highlighting the importance of cultural differences in this area.

The productivity losses of the informal caregivers varied for the different diseases and were likely influenced by a number of factors (figure 1). The different patterns regarding the number of days lost in the ICU for the caregivers relative to the patients for tetanus cases could be influenced by the fact that a higher proportion of tetanus patients did not live in HCMC; thus, their family members would have to travel a further distance.

It should be noted that we used the opportunity cost approach to value the informal caregiver’s unpaid work, which values time spent on unpaid work in terms of the value of the next best alternative activity the individual has forgone in order to perform it.^43^,^46^ The alternative replacement cost approach, which measures the value of time spent on unpaid work based on what it would cost to hire a paid worker to perform the same tasks, could have yielded different estimates.^43^

Beyond the economic burden incurred by these informal caregivers presented here, it is important to note that the caregivers would suffer a substantial impact on their mental health and quality of life.^47 48^ Thus, further research is needed to fully capture the burden on caregivers.

### The incurred direct non-medical costs

The average direct non-medical cost was US$169.58 per patient, in which US$74.59 was incurred by the patient and US$90.89 for their caregivers. The costs related to the food for the patients while in ICU were mostly negligible. This was because the majority of the patients were prescribed nutritional supplements during their time in ICU—which were considered a direct medical cost and not included. The costs related to food for informal caregivers were projected based on their reported daily average amount spent. In practice, the food costs incurred by informal caregivers are difficult to estimate accurately. For example, many informal caregivers shared food with others, many received free food from a charity or many had other family members bring it to them.

The majority of the patients (73.40%) used an ambulance on the day they were admitted to the hospital. The corresponding travel-related cost was heavily influenced by whether they were a resident of HCMC or not (online supplemental figure S1). It should be noted that the majority of patients transferred from other hospitals still have to pay for their ambulance, even if they are insured (by the governmental health insurance programme) and follow a recognised pathway of care.^49^ This is because only some special groups of patients have their ambulance cost covered following health insurance law.^49^ Thus, the range of travel costs incurred by patients was significantly higher for those who lived in other southern and central highland provinces.

In our sample, almost all the informal caregivers stayed in the hospital to take care of the patients during their hospitalisation. The hospital charged less than US$0.50 per day for hospital accommodation for the caregivers while their family members stayed in the ICU. After the patients were transferred to the general ward, the caregivers could stay with them and were not charged. Thus, for this setting, the accommodation costs for caregivers were relatively insignificant. It should be noted that the free accommodation provided for one caregiver accompanying a hospitalised patient is commonly applied across governmental hospitals in Vietnam.

### Indirect costs and valuing unpaid care work

The indirect costs and valuation of informal care were key drivers of the estimated burden of these non-medical costs. The total indirect cost estimates varied between US$242.34 and US$541.02, depending on the approach taken. Some of this variation was caused by the chosen wage source. Beyond this, a further source of variation was related to if the informal caregivers’ time was valued based on a daily wage or an hourly basis.^37 45 50^ This caused the average indirect costs related to the informal caregivers to vary between US$159.83 and US$274.51 per patient (scenario 2 vs scenario 3).

A high proportion of our sample of patients and caregivers were not in formal employment. It is important to note that in this context, using valuation approaches that use reported wages of the sample potentially values the productivity losses of those in formal employment lower than others in the population. This highlights the importance of considering equity issues in the valuation of indirect costs.^34^

Further guidance is needed on the valuation of informal care.^24 25 44 51^ Krol and Brouwer^44^ highlighted that little guidance is offered regarding the inclusion of unpaid labour in economic evaluations. This is an area that needs further development and is a particularly pertinent issue related to the costs of critically ill patients. Hubens *et al*^51^ found unpaid work is often not included in the measurement instruments of productivity loss that they identified within a systematic review. Regarding developments in this area, it is important to consider gender inequality in caregiving roles.^52^ Urwin *et al*^24^ also highlighted that the measurement of informal care is important to include within economic evaluations but there is little consensus on how to appropriately measure this type of care.

### Policy implications

UHC is a priority for the Vietnamese government. Vietnam’s social health insurance programme was first introduced in 1992 with the aim of preventing impoverishment of patients due to healthcare expenditure.^53^ The patient’s copayment rate for healthcare services is typically 20% if they follow the correct referral pathway (this rate is lower for certain subsidised groups).^54^ By the end of 2023, the programme’s coverage was 93.35% of the total population.^55^ Although the national health coverage is high, patients can still incur high out-of-pocket healthcare costs.^56 57^

These cost estimates highlight the need to reduce the costs of critical care in settings like Vietnam, particularly in the context of targets of universal health coverage. Importantly, even critically ill patients with the highest level of coverage from the national health insurance programme still had significant costs.^10^ The significance of the full cost of critical care also has implications for countries’ health insurance policies, such as copayment rates, coverage of costs related to the use of ambulances and policies related to waiting periods for eligibility after individuals register or renew their health insurance. More broadly, this work highlights the importance of considering the full picture of the costs and the potential importance of valuing the costs.

Informal caregivers also experienced considerable productivity losses. There is legislation in Vietnam to support caregivers who are caring for certain vulnerable groups or those living with certain disabilities. However, there is currently no legislation to support informal caregivers for hospitalised patients. Accordingly, to reduce the financial burden on these caregivers, policies that support or subsidise caregivers who need to take leave from work to care for hospitalised patients should be considered. Besides financial issues, Long *et al*^30^ found that even moderate social support was positively associated with the key coping styles of Vietnamese caregivers and thus can enhance their quality of life. Hence, further research is needed to quantify the burden on informal caregivers and policies/measures to support them.

### Limitations

The study used a convenience sampling approach which is discussed in the “Generalisability and representativeness of the sample” subsection. Our sample for sepsis was lower compared with the other diseases.

As we were not able to follow up patients after hospital discharge, it is likely that significant additional costs were incurred, particularly by those families and patients in the tetanus and sepsis groups in whom long-term disability has been shown in this setting.^58^

It should be noted that this analysis only focused on the costs incurred during the time that the patients were admitted to the HTD in HCMC. However, patients could have been transferred from lower level hospitals before being admitted to the HTD. Thus, this study would not capture the full non-medical costs related to these cases. These factors might contribute to the underestimation of the economic burden of both patients and their caregivers.

A further limitation was that it was not possible to collect daily information from the patients—particularly while they were in the ICU. Some of the questions would therefore be subject to recall bias. Furthermore, the questions regarding transportation costs focused on the day the patient and/or informal caregivers arrived at the hospital and the day they left the hospital (table 1). Thus, it was not possible to capture the costs of any additional transportation if informal caregivers travelled back and forth to the hospital multiple times. In addition, the transportation costs for those using their own transportation (ie, their motorbike) were not fully captured. Consequently, the transportation cost might be underestimated. In addition, the costs related to other types of non-medical resources were not captured (such as costs related to clothing, electrical fans etc.).

The food-related cost was difficult to estimate accurately, and some patients/informal caregivers received free food from the hospital but could not remember exactly the number of times they received it or when they received free food from different sources such as vouchers from the canteen. That said, there was little variation in food cost per day across diseases.

It should be noted that some food and transportation costs would have been incurred for normal day-to-day living if the patient was not hospitalised. However, the majority of our sample lived outside HCMC, and their routine expenses would likely be much smaller than what was incurred due to the hospitalisation episode. Thus, this is unlikely to have notably influenced our results.

Finally, the indirect costs were estimated based on monetising the number of days lost by the patients/caregivers rather than specifically the number of workdays lost. It could be argued that this could overestimate the corresponding indirect costs. To account for this, a range of different scenarios was used for the valuation of productivity losses.

## Conclusion

This study demonstrates that critically ill tetanus, sepsis and dengue patients and their families can incur notable direct and indirect non-medical costs. It is important to note that the data collected were limited to one hospital and a convenience sampling approach was used to enrol patients. Consequently, the results should not be overgeneralised, and it is important that further studies are conducted in this area. Despite this, it highlights the importance of not only accounting for direct medical costs when considering the full costs for critically ill patients. This is particularly relevant in the context of UHC targets, as even with 100% coverage of their medical costs, many families are still likely to suffer significant financial hardship. This also emphasises the importance of valuing unpaid work and informal care, particularly in the context of critical care.

## Supplementary material

10.1136/bmjph-2024-002169 online supplemental file 1

## Data Availability

Data are available upon reasonable request.
