# Supplementary material for: Non-medical costs incurred by critically ill patients with dengue, sepsis and tetanus within a major referral hospital in Southern Vietnam: a cost of illness study
Source: BMJ Public Health. 2025 Aug 20;3(2):e002169. doi: 10.1136/bmjph-2024-002169 (PMC12366576; doi:10.1136/bmjph-2024-002169)
Supplement: online supplemental file 1 [file bmjph-3-2-s001.docx]

**Supplementary Materials**

| **Box S1: Minimum wage**  The minimum wage in Vietnam varies across the different provinces. There are four different groupings:  Area 1: Ho Chi Minh, Binh Duong, Dong Nai, Vung Tau, Can Tho.  Area 2: Tay Ninh.  Area 3: Binh Thuan, Binh Phuoc, Long An, Tien Giang, An Giang, Kien Giang, Ca Mau, Ben Tre, Kon-Tum, Bac Lieu, Lam Dong, Khanh Hoa, Hau Giang.  Area 4: Tra Vinh, Soc Trang, Dak Nong, Dak Lak, Dong Thap, Gia Lai.  The following monthly minimum wages (US$140, US$157, US$180, US$202), daily minimum wages (US$7.78, US$6.91, US$6.04, US$5.40), and hourly minimum wages (US$0.97, US$0.86, US$0.76, US$0.67) correspond to areas 1,2,3, and 4, respectively. These are based on the assumption that a person works 8 hours a day and 26 days a month.  When estimating the indirect costs, we used the minimum wage that corresponded to the address (province) of each patient. The informal caregivers were assumed to live in the same area as the patient |
| --- |

| **Table S1: Indirect cost valuations approaches** | |
| --- | --- |
| **Scenario 1:** All productivity losses valued based on the daily minimum wage. | The productivity losses of both the patients and their caregivers were valued based on Vietnam’s minimum wage. The median daily minimum wage for this sample was estimated to be US$7.77^α^ (based on the average monthly minimum wage divided by 26 days^β^). This was multiplied by the number of days they spent at the hospital. |
| **Scenario 2:** Productivity losses for patients and caregivers in full-time employment valued based on their reported wages. For those not in full-time employment, productivity losses were valued based on the daily minimum wage. | For the patients and caregivers: The productivity losses for those in full-time employment were valued based on a daily value approximated from their reported wage. The median daily wage reported for these patients and caregivers were US$11.72 and US$12.90 respectively (based on their reported monthly income assuming 26 working days per month)^β^.  The productivity losses for those not in full-time employment or for those for whom wage data was not available, were valued based on a daily value approximated from Vietnam’s minimum wage (converted into a daily value). The median daily minimum wage for this sample was estimated to be US$7.77^α^ (based on the average monthly minimum wage divided by 26 days^β^. This was multiplied by the number of days they spent at the hospital.  Within this scenario, unpaid work was being valued inline with the opportunity cost approach^1^. |
| **Scenario 3:** Productivity losses for patients in full-time employment were valued based on their reported wages. For those not in full-time employment, the minimum wage was used. The productivity losses of the caregivers were valued based on an hourly minimum wage (considering each hour they spent with patient). | The productivity losses were valued differently for patients and caregivers.  Patients: For patients in full-time employment their productivity losses were valued based on their reported wages. The median daily wage for these patients was estimated to be US$11.72 (based on their reported monthly wage divided by the number of working days reported per week multiple by 4.3 weeks). For those not in full-time employment or for those for whom wage data was not available, the productivity losses were valued based on Vietnam’s minimum wage (converted into a daily value). The average daily minimum wage for this sample was estimated to be US$7.77^α^ (based on the average monthly minimum wage divided by 26 days^β^). These were multiplied by the number of days they were at the hospital.  Informal caregivers: The productivity losses of the caregivers were valued based on the hourly minimum wage multiplied by the number of hours they spent with the patient at the hospital. The average minimum wage per hour for this sample was assumed to be US$0.97. |
| **Scenario 4:** Productivity losses valued based on the per capita GDP for both patients and informal caregivers. | The productivity losses of both the patients and caregivers were valued based on the daily per capita GDP for Vietnam. Daily per capita GDP was estimated to be US$14.45 (estimated by dividing the annual per capita GDP (US$3,756.5^2^) by 260 working days^¥^). This value was multiplied by the number of days the patient and caregivers spent at the hospital. |
| **Scenario 5:** Productivity losses for those in formal employment valued based on the average national wage and productivity losses for those not in formal employment were valued based on the daily minimum wage. | The productivity losses were valued differently depending on the patients’ and caregivers’ employment status.  The productivity losses for those in formal employment were valued based on an approximated value of daily paid work. This was estimated to be US$13.06 per day (based on the overall average monthly wage for Vietnam reported by the General Statistics Office of Vietnam ^3^ (US$282.90 divided by 21.67 days^¥^).  The productivity losses for those not in formal employment were valued based on the average daily minimum wage. The average daily minimum wage was estimated to be US$6.53^α^ (based on the average monthly minimum divided by 26^β^).  These values were multiplied by the number of days the patient/caregiver spent at the hospital. |
| ^α^ *The minimum wage in Vietnam varies across the different provinces. The values reported here are averages based on the address of the patients (see Supplementary Box S1). All costs were calculated in Vietnam Dong and then converted to US Dollars with 1 US$ = 23160 VND ^4^.*  *^β^ 26 work days per month with 8 working hours a day are based on the assumptions used within the government’s minimum wage calculations ^5^. This is consistent with the average number of working days reported by our sample (25.8).*  *^¥^ 21.67 work days per month is based on the report by Vietnam’s General Statistics Office in 2022 ^3^, which stated that on average a person works 40.3 hours a week with an average 4.3 weeks a month.* | |

| **Table S2: Valuation of the daily productivity losses** | | |
| --- | --- | --- |
|  | **Patient** | **Caregiver** |
| Scenario 1: All productivity losses valued based on the minimum wage. | Median: US$7.77  Mean: US$6.95 | Median: US$7.77  Mean: US$6.94 |
| Scenario 2: Productivity losses for patients and caregivers in full-time employment valued based on their reported wages. For those not in full-time employment, productivity losses were valued based on the daily minimum wage. | Median: US$8.95  Mean: US$8.69 | Median: US$7.77  Mean: US$10.21 |
| Scenario 3: Productivity losses for patients in full-time employment were valued based on their reported wages. For those not in full-time employment, the minimum wage was used. The productivity losses of the caregivers were valued based on an hourly minimum wage (considering each hour they spent with patient). | Median: US$8.95  Mean: US$8.69 | Median: US$23.32  Mean: US$20.83 |
| Scenario 4: Productivity losses valued based on the per capita Gross Domestic Product (GDP) for both patients and informal caregivers. | Median: US$14.45  Mean: US$14.45 | Median: US$14.45  Mean: US$14.45 |
| Scenario 5: Productivity losses for those in formal employment valued based on the average national wage and productivity losses for those not in formal employment were valued based on the daily minimum wage. | Median: US$6.53  Mean: US$8.96 | Median: US$8.81  Mean: US$9.26 |
| *See Supporting Table S1 for a more detailed description of the different scenarios.* | | |

| **Table S3: Basic characteristics of the patients’ informal caregivers** | | | | | |
| --- | --- | --- | --- | --- | --- |
|  | | **Dengue (n=97)** | **Sepsis  (n=33)** | **Tetanus (n=74)** | **Overall (n=204)** |
| **Gender** | |  |  |  |  |
| Female | | 56 (57.73%) | 18 (54.55%) | 42 (56.76%) | 116 (56.86%) |
| Male | | 41 (42.27%) | 15 (45.45%) | 32 (43.24%) | 88 (43.14%) |
| **Age (years)** | |  |  |  |  |
| Median [Q1, Q3] | | 41.00  [31.00; 47.00] | 42.00  [33.00; 47.00] | 41.00  [36.00; 51.00] | 41.00  [33.00; 50.00] |
| **Relationship to patient** | |  |  |  |  |
| Child | | 17 (17.53%) | 17 (51.52%) | 45 (60.81%) | 79 (38.73%) |
| Parent | | 28 (28.87%) | 4 (12.12%) | 4 (5.41%) | 36 (17.65%) |
| Spouse | | 14 (14.43%) | 7 (21.21%) | 15 (20.27%) | 36 (17.65%) |
| Other relative | | 11 (11.34%) | 1 (3.03%) | 2 (2.70%) | 14 (6.96%) |
| Sibling | | 24 (24.74%) | 4 (12.12%) | 7 (9.46%) | 35 (17.16%) |
| Other | | 3 (3.09%) | 0 (0%) | 1 (1.35%) | 4 (1.96%) |
| **Employment** | |  |  |  |  |
| Full time job | | 34 (35.05%) | 13 (39.39%) | 22 (29.73%) | 69 (33.82%) |
| Looking after home | | 11 (11.34%) | 8 (24.24%) | 12 (16.22%) | 31 (15.20%) |
| Own business/farming | | 32 (32.99%) | 4 (12.12%) | 24 (32.43%) | 60 (29.41%) |
| Part time job | | 2 (2.06%) | 1 (3.03%) | 4 (5.41%) | 7 (3.43%) |
| Retired | | 1 (1.03%) | 3 (9.09%) | 5 (6.76%) | 9 (4.41%) |
| Student | | 4 (4.12%) | 0 (0%) | 1 (1.35%) | 5 (2.45%) |
| Unemployed | | 2 (2.06%) | 2 (6.06%) | 0 (0%) | 4 (1.96%) |
| Other | | 11 (11.34%) | 2 (6.06%) | 6 (8.11%) | 19 (9.31%) |
| **Method of transportation to the hospital** | | | |  |  |
| Ambulance | | 44 (45.36%) | 16 (48.48%) | 34 (45.95%) | 94 (46.08%) |
| Bus | | 15 (15.46%) | 3 (9.09%) | 19 (25.68%) | 37 (18.14%) |
| Hired motorbike | | 3 (3.09%) | 3 (9.09%) | 1 (1.35%) | 7 (3.43%) |
| Own motorbike | | 28 (28.87%) | 11 (33.34%) | 9 (12.16%) | 48 (23.53%) |
| Taxi/hired bike | | 6 (6.19%) | 0 (0%) | 8 (10.81%) | 14 (6.86%) |
| Other | | 1 (1.03%) | 0 (0%) | 3 (4.05%) | 4 (1.96%) |
| **Travelled with the patient on admission day** | | | |  |  |
| No | | 40 (41.24%) | 16 (48.48%) | 28 (37.84%) | 84 (41.18%) |
| Yes | | 57 (58.76%) | 17 (51.52%) | 46 (62.16%) | 120 (58.82%) |
| **Total number of hours spent a day at the ICU** | | |  |  |  |
| Mean (SD) | 17.27 (8.74) | | 20.33 (7.22) | 19.64 (8.50) | 18.63 (8.49) |
| **Total number of hours spent a day at the general wards** | | | |  |  |
| Mean (SD) | 14.49 (10.45) | | 11.42 (11.37) | 15.22 (10.97) | 14.25 (10.82) |
| **Total number of days spent at the ICU** | | |  |  |  |
| Median [Q1; Q3] | | 5.04  [4.00; 10.25] | 13.83  [6.75; 34.50] | 20.00  [11.00; 29.50] | 11.00  [5.00; 25.75] |
| **Total number of days spent at the general ward** | | | |  |  |
| Median [Q1; Q3] | | 4.42  [2.00; 8.55] | 8.50  [6.0; 16.24] | 6.50  [3.00; 9.75] | 5.10  [2.02; 10.00] |
| **Total number of days spent at the hospital** | | |  |  |  |
| Median [Q1; Q3] | | 9.83  [7.57; 21.50] | 29.15  [15.69; 44.25] | 25.00  [18.15; 37.19] | 19.00  [9.72; 34.00] |
| **Number of different caregivers whilst patient in the ICU** | | | | |  |
| Median [Q1; Q3] | | 2.00  [2.00; 3.00] | 4.00  [2.0;0 4.00] | 2.00  [1.00; 3.00] | 2.00  [2.00; 3.00] |
| **Number of different caregivers whilst patient in the general ward** | | | | |  |
| Median [Q1, Q3] | | 2.00  [1.00, 2.50] | 2.00  [1.00, 3.00] | 2.00  [1.00, 2.50] | 2.00  [1.00, 3.00] |
| *Q1: 25% of interquartile range and Q3: 75% of interquartile range; SD: Standard deviation* | | | | | |

| **Table S4: The non-medical cost of both patients and informal caregivers** | | | | |
| --- | --- | --- | --- | --- |
|  | **Dengue (n=44)** | **Sepsis  (n=12)** | **Tetanus (n=38)** | **Overall (N=94)** |
| **Total non-medical cost (both patient and caregivers)** | | | | |
| Mean (SD) | 196.13 (183.08) | 256.78 (198.16) | 298.49 (198.22) | 245.25 (195.17) |
| Median [Q1; Q3] | 130.48  [83.44; 277.96] | 225.82  [128.05; 350.89] | 235.86  [146.55; 398.21] | 169.58 [104.44; 362.37] |
| **Total food cost (both patient and caregivers)** | | | | |
| Mean (SD) | 102.79 (78.95) | 138.95 (131.81) | 142.36 (114.20) | 123.40 (102.49) |
| Median [Q1; Q3] | 90.67  [48.56; 125.69] | 119.00  [37.05; 202.94] | 106.22  [68.38; 175.95] | 97.58  [58.68; 167.04] |
| **Total transportation cost (both patient and caregivers)** | | | | |
| Mean (SD) | 97.05 (143.42) | 126.24 (195.97) | 173.64 (178.67) | 131.74 (167.47) |
| Median [Q1; Q3] | 41.02  [6.26; 114.64] | 24.18  [6.67; 167.85] | 120.36  [49.22; 231.54] | 79.02  [13.44; 187.82] |
| *Q1: 25% of interquartile range and Q3: 75% of interquartile range; SD: Standard deviation* | | | | |

**Table S5: Total indirect costs of patients and informal caregivers across the different calculation scenarios – stratified by disease.**

|  | **Dengue (n=44)** | **Sepsis (n=12)** | **Tetanus (n=38)** | **Overall (n=94)** |
| --- | --- | --- | --- | --- |
| **Scenario 1** | |  |  |  |
| Patients | 62.18  [46.63; 93.26] | 137.31  [99.09; 256.26] | 178.76  [116.36; 229.71] | 105.79  [62.17; 185.45] |
| Caregivers | 66.71  [51.10; 164.72] | 206.82  [106.16; 343.91] | 174.01  [109.24; 226.68] | 120.90  [66.40; 211.57] |
| **Scenario 2** | |  |  |  |
| Patients | 72.75  [48.52; 107.59] | 163.21  [101.04; 332.25] | 185.77  [128.45; 312.18] | 120.70  [77.72; 227.93] |
| Caregivers | 102.42  [61.79; 179.94] | 346.11  [145.73; 519.66] | 188.15  [143.44; 257.32] | 159.83  [94.12; 246.70] |
| **Scenario 3** | |  |  |  |
| Patients | 72.75  [48.52; 107.59] | 163.21  [101.04; 332.25] | 185.77  [128.45; 312.18] | 120.70  [77.720; 227.93] |
| Caregivers | 200.13  [153.30; 494.17] | 620.47  [318.49; 1031.74] | 522.12  [327.72; 680.05] | 362.69  [199.16; 634.72] |
| **Scenario 4** | |  |  |  |
| Patients | 137.26  [97.53; 223.95] | 288.96  [198.66; 487.62] | 368.43  [263.68; 538.19] | 245.62  [144.48; 415.38] |
| Caregivers | 142.1  [109.41; 310.63] | 421.10  [226.65; 639.33] | 361.20  [262.17; 537.29] | 274.51  [140.42; 491.23] |
| **Scenario 5** | |  |  |  |
| Patients | 85.12  [60.48; 138.88] | 179.20  [123.20; 302.40] | 228.48  [163.52; 333.76] | 152.32  [89.60; 257.60] |
| Caregivers | 91.06  [70.13; 199.09] | 269.89  [145.27; 409.76] | 231.50  [168.03; 344.36] | 175.94  [90.00; 314.84] |

| **Table S6: Indirect costs of the patients and informal caregivers across different scenarios (2021 US$ prices)** | | | |
| --- | --- | --- | --- |
| **Scenarios** | **Indirect cost: Patient** | **Indirect cost: Caregivers** | **Total indirect cost** |
| Scenario 1: All productivity losses valued based on the minimum wage. | US$105.79  (62.17–185.45) | US$120.90  (66.40–211.57) | US$242.34 (145.83–428.08) |
| Scenario 2: Productivity losses for patients and caregivers in full time employment valued based on their reported wages. For those not in full time employment, productivity losses were valued based on the daily minimum wage. | US$120.70  (77.72–227.93) | US$159.83  (94.12–246.70) | US$302.25 (176.40–529.12) |
| Scenario 3: Productivity losses for patients in full time employment were valued based on their reported wages. For those not in full time employment, the minimum wage was used. The productivity losses of the caregivers were valued based on an hourly minimum wage (considering each hour they spent with patient). | US$120.70  (77.72–227.93) | US$362.69  (199.16–634.72) | US$ 541.02 (291.72–913.41) |
| Scenario 4: Productivity losses valued based on the per capita GDP for both patients and informal caregivers. | US$245.62  (144.48–415.38) | US$274.51  (140.42–491.23) | US$534.58 (288.96–892.17) |
| Scenario 5: Productivity losses for those in formal employment valued based on the average national wage and productivity losses for those not in formal employment were valued based on the daily minimum wage. | US$152.32  (89.60–257.60) | US$175.94  (90.00–314.84) | US$337.07  (182.20–562.81) |
| *Further description related to the different scenarios for the valuation of the indirect costs is presented in Table S1.* | | | |

**Table S7:** **The total direct/indirect non-medical cost per day across indirect cost calculation scenarios**

|  | **Dengue (N=44)** | **Sepsis (N=12)** | **Tetanus (N=38)** | **Overall (N=94)** |
| --- | --- | --- | --- | --- |
| **Total cost per day (Scenario 1)** | |  |  |  |
| Mean (SD) | 34.85 (20.49) | 31.05 (14.65) | 25.68 (10.58) | 30.66 (16.80) |
| Median [Q1; Q3] | 28.90  [24.50; 38.45] | 27.70  [20.92; 32.90] | 21.70  [19.63; 29.76] | 27.67  [20.48; 33.57] |
| **Total cost per day (Scenario 2)** | |  |  |  |
| Mean (SD) | 39.43 (22.91) | 42.17 (28.56) | 30.26 (16.41) | 36.07 (21.69) |
| Median [Q1; Q3] | 32.92  [24.50; 48.75] | 34.06  [24.87; 46.96] | 22.18  [20.54; 35.67] | 29.55  [21.89; 43.99] |
| **Total cost per day (Scenario 3)** | |  |  |  |
| Mean (SD) | 54.83 (27.57) | 53.53 (18.37) | 42.62 (21.60) | 49.73 (24.74) |
| Median [Q1; Q3] | 48.79  [40.04; 60.37] | 49.64  [36.65; 67.09] | 37.45  [32.42; 48.46] | 44.71  [34.81; 55.01] |
| **Total cost per day (Scenario 4)** | |  |  |  |
| Mean (SD) | 51.97 (23.97) | 47.96 (16.00) | 42.12 (14.33) | 47.47 (19.98) |
| Median [Q1; Q3] | 46.01  [38.11; 54.85] | 47.21  [34.35; 53.17] | 37.74  [34.69; 47.01] | 43.45  [35.29; 52.23] |
| **Total cost per day (Scenario 5)** | |  |  |  |
| Mean (SD) | 39.53 (21.66) | 35.57 (15.36) | 30.88 (11.56) | 35.53 (17.73) |
| Median [Q1; Q3] | 33.48  [28.04; 43.78] | 32.75  [23.61; 39.80] | 27.04  [24.06; 34.64] | 31.69  [24.63; 38.03] |
|  |  |  |  |  |
| *The scenarios refer to the approach taken for the indirect cost calculation (as defined in Table S1)* | | | | |

**Table S8: The multiple linear regression analysis between total cost and important variables**

| Scenario | Intercept  (95% CI) | Gender (95% CI) (References: Male) | Address (95% CI)  (Reference: Ho Chi Minh City) | ICU days (95% CI) | General ward days (95% CI) | Number of caregivers (95% CI) | Adjusted R2 |
| --- | --- | --- | --- | --- | --- | --- | --- |
| Scenario 1 | 158.615 (120.519-208.754) **** | 0.993 (0.813-1.214) | 1.404 (1.141-1.729) ** | 1.024 (1.017-1.029) **** | 1.069 (1.041-1.097) **** | 1.047 (0.959-1.144) | 0.56 **** |
| Scenario 2 | 183.422 (133.845-251.362) **** | 0.978 (0.777-1.231) | 1.510 (1.19-1.917) **** | 1.024 (1.017-1.031) *** | 1.061 (1.03-1.093) **** | 1.043 (0.943-1.155) | 0.49 **** |
| Scenario 3 | 248.806 (186.758-331.469) **** | 1.047 (0.849-1.291) | 1.293 (1.041-1.607) * | 1.023 (1.017-1.03) **** | 1.068 (1.04-1.098) **** | 1.084 (0.989-1.189) | 0.53 **** |
| Scenario 4 | 232.489 (181.181-298.326) **** | 1.031 (0.860-1.237) | 1.392 (1.152-1.681) *** | 1.027 (1.022-1.033) **** | 1.068 (1.043-1.094) **** | 1.059 (0.978-1.148) | 0.66 **** |
| Scenario 5 | 170.778 (131.144-222.392) **** | 1.010 (0.833-1.225) | 1.495 (1.224-1.826) *** | 1.025 (1.02-1.031) **** | 1.069 (1.043-1.097) **** | 1.053 (0.967-1.146) | 0.62 **** |
| ** <0.05; **<0.01; ***<0.001, ****<0.0001*  *CI: Confidence Interval*  *Address is a dichotomous variable including two subgroups’ patients living in Ho Chi Minh City and those living outside Ho Chi Minh City. People living in Ho Chi Minh City and Male are references*  *The scenarios refer to the approach taken for the indirect cost calculation (as defined in Table S1)* | | | | | | | |

| **Table S9: Reported financial coping strategies** | | | | |  |
| --- | --- | --- | --- | --- | --- |
|  | **Dengue (n=44)** | **Sepsis  (n=12)** | **Tetanus (n=38)** | **Overall (N=94)** | |
| **Coping strategies** |  |  |  |  | |
| Private loan | 3 (6.82%) | 0 (0%) | 1 (2.63%) | 4 (4.26%) | |
| Borrowing family/friends | 22 (50.00%) | 4 (33.33%) | 22 (57.89%) | 48 (51.06%) | |
| Did not need to borrow money | 16 (36.36%) | 8 (66.67%) | 14 (36.84%) | 38 (40.43%) | |
| Selling assets | 3 (6.82%) | 0 (0%) | 0 (0%) | 3 (3.19%) | |
| None reported | 0 (0%) | 0 (0%) | 1 (2.64%) | 1 (1.06%) | |
| **Average money borrowed** | |  |  |  | |
| Mean (SD) | 1,864.09 (2,101.76) | 1,943.01 (431.78) | 1,514.82 (955.52) | 1,722.57 (1,620.50) | |
| Median [Q1, Q3] | 1,036.27  [863.56; 2,158.89] | 2,158.89  [1,943.01; 2,158.89] | 1,295.34  [863.56; 2,158.89] | 1,295.34  [863.56; 2,158.89] | |
| *Q1: 25% of interquartile range and Q3: 75% of interquartile range.*  *Values in 2021 prices.* | | | | | |

| **Table S10: Household characteristics** | | | | |
| --- | --- | --- | --- | --- |
|  | **Dengue (n=44)** | **Sepsis  (n=12)** | **Tetanus (n=38)** | **Overall (N=94)** |
| **Number of members within a household** | |  |  |  |
| Median [Q1; Q3] | 4.00  [3.00; 5.00] | 4.00  [2.00; 5.00] | 3.00  [2.00; 4.00] | 4.00  [2.25; 5.00] |
| Mean (SD) | 4.14 (2.03) | 4.00 (2.41) | 3.29 (1.71) | 3.78 (1.98) |
| **Average household income per month (US$)** | |  |  |  |
| Median [Q1; Q3] | 647.67  [431.78; 1036.27] | 723.23  [388.60; 1051.38] | 496.55  [323.83; 647.67] | 604.49  [388.60; 863.56] |
| Mean (SD) | 734.40 (420.62) | 870.57 (694.71) | 545.20 (318.46) | 673.61 (434.66) |
| **Average household expenditure per month (US$)** | |  |  |  |
| Median [Q1; Q3] | 474.96  [302.25; 820.38] | 539.72  [323.83; 782.60] | 302.25  [140.33; 431.78] | 431.78  [226.68; 647.67] |
| Mean (SD) | 597.53 (398.56) | 596.39 (372.49) | 318.26 (197.08) | 473.72 (345.71) |
| *Q1: 25% of interquartile range and Q3: 75% of interquartile range.* | | | | |


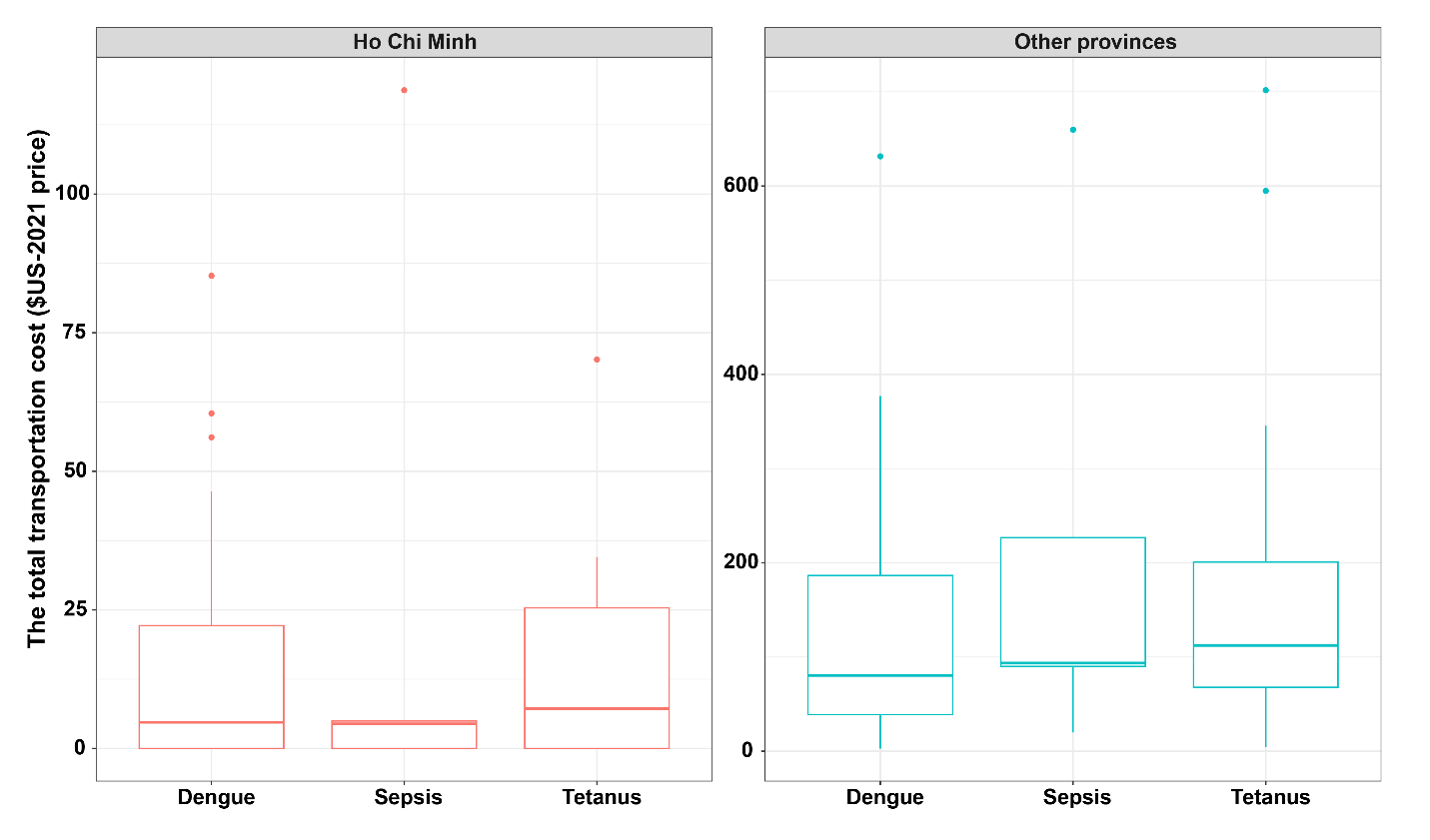


**Figure S1: The transportation cost incurred by the patients stratified by place of residence.**


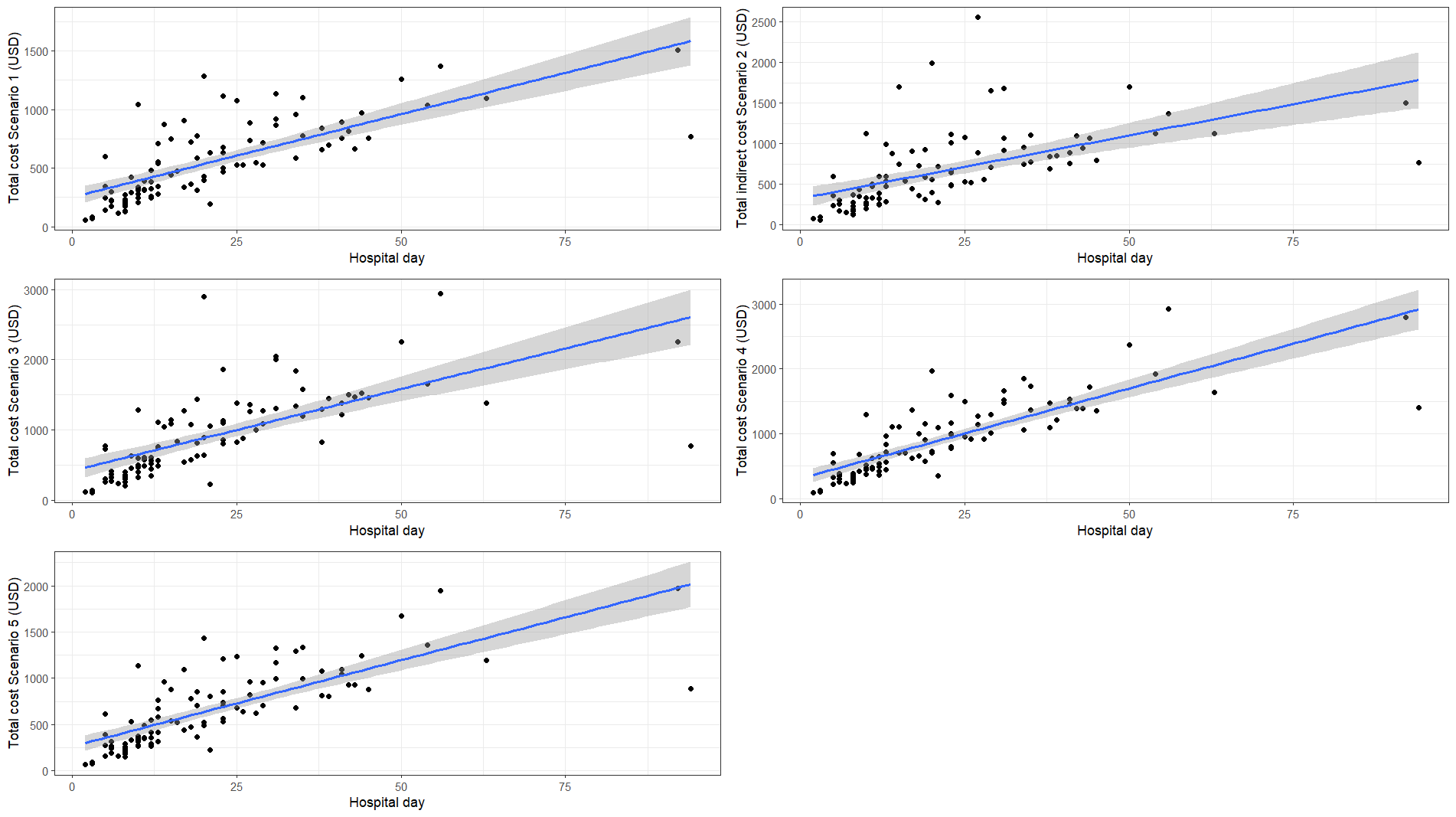


**Figure S2: The association between total cost and the length of stay in the hospital**


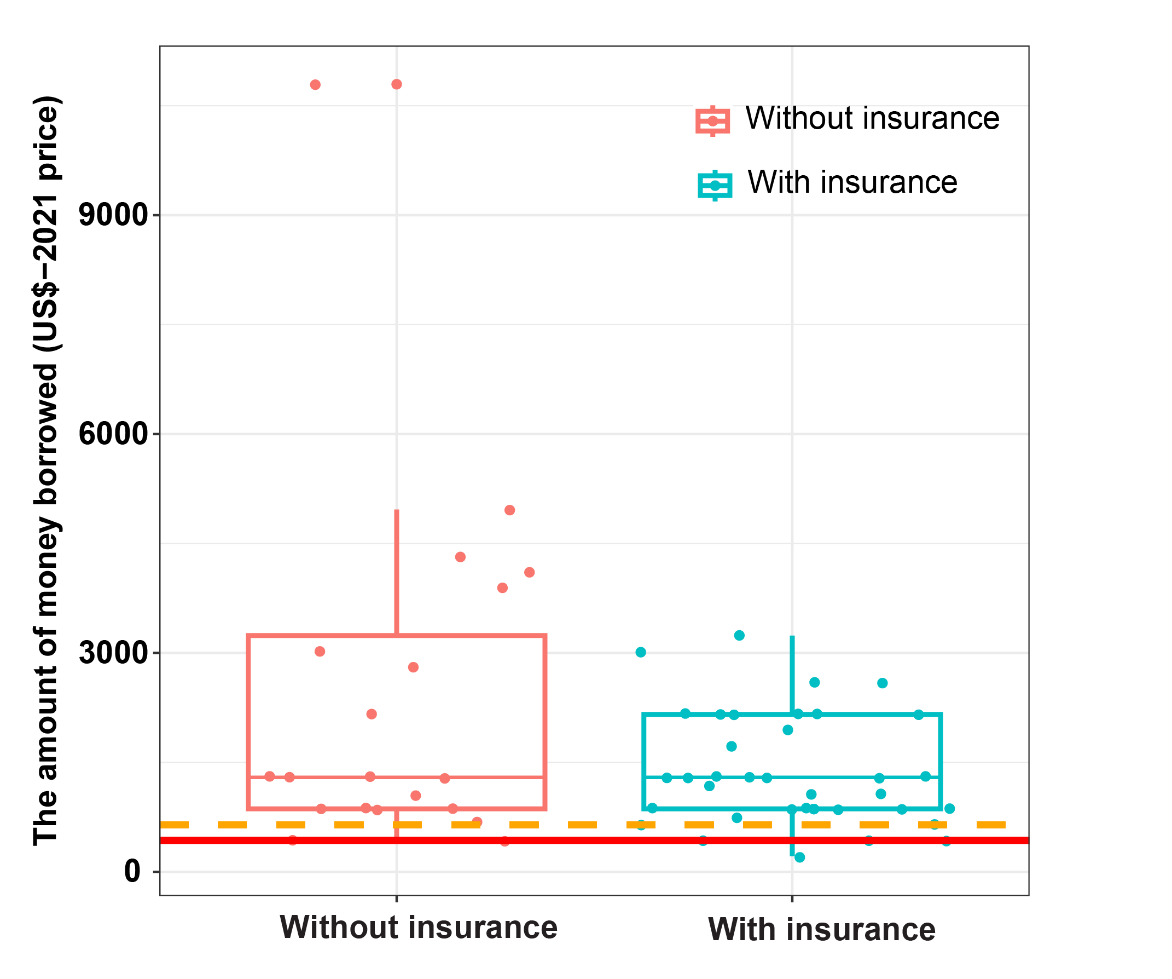


**Figure S3: The average amount of money borrowed.** *Note that this is the average for those that borrowed money and not the average for the whole sample***.** *The box plot illustrates the interquartile range. The red line represents the reported household expenditure and orange dashed line represents the reported household income (Table 3 of the main text).*

**References**

1. Krol M, Brouwer W, Rutten F. Productivity costs in economic evaluations: past, present, future. *Pharmacoeconomics* 2013;31(7):537-49. doi: 10.1007/s40273-013-0056-3

2. Bank W. GDP per capita (current US$) - Vietnam 2023 [cited 2022 25 Jan]. Available from: <https://data.worldbank.org/indicator/NY.GDP.PCAP.CD?locations=VN> accessed 25th Jan 2023.

3. General Statistics Office. Report on labor force survey 2021. Hanoi, 2022.

4. World Bank. Official exchange rate - Vietnam 2022 [27/12/2022]. Available from: <https://data.worldbank.org/indicator/PA.NUS.FCRF?locations=VN> accessed 27/12/2022 2022.

5. The Vietnam Government. Decree 38/2022/ND-CP Prescribing Statutory Minimum Wages Paid To Employees Working Under Employment Contracts. Hanoi: Vietnam government, 2022.
